# Asteltoxin inhibits extracellular vesicle production through AMPK/mTOR-mediated activation of lysosome function

**DOI:** 10.1038/s41598-022-10692-0

**Published:** 2022-04-23

**Authors:** Fumie Mitani, Jianyu Lin, Tatsuya Sakamoto, Ryo Uehara, Tomoya Hikita, Takuya Yoshida, Andi Setiawan, Masayoshi Arai, Chitose Oneyama

**Affiliations:** 1 Division of Cancer Cell Regulation, Aichi Cancer Center Research Institute, Chikusa-ku, Nagoya, Japan; 2 Department of Oncology, Graduate School of Pharmaceutical Sciences, Nagoya City University, Mizuho-ku, Nagoya, Japan; 3 Laboratory of Natural Products for Drug Discovery, Graduate School of Pharmaceutical Sciences, Osaka University, Suita, Osaka Japan; 4 Department of Target and Drug Discovery, Graduate School of Medicine, Nagoya University, Showa-ku, Nagoya, Japan; 5 Laboratory of Biophysical Chemistry, Graduate School of Pharmaceutical Sciences, Osaka University, Suita, Osaka Japan; 6 Department of Chemistry, Faculty of Mathematics and Natural Sciences, Lampung University, Bandar Lampung, Indonesia; 7 JST, PRESTO, Nagoya, Japan

**Keywords:** Cell signalling, Drug screening, Natural products, Cancer

## Abstract

Cancer cells secrete aberrantly large amounts of extracellular vesicles (EVs) including exosomes, which originate from multivesicular bodies (MVBs). Because EVs potentially contribute to tumor progression, EV inhibitors are of interest as novel therapeutics. We screened a fungal natural product library. Using cancer cells engineered to secrete luciferase-labeled EVs, we identified asteltoxin, which inhibits mitochondrial ATP synthase, as an EV inhibitor. Low concentrations of asteltoxin inhibited EV secretion without inducing mitochondrial damage. Asteltoxin attenuated cellular ATP levels and induced AMPK-mediated mTORC1 inactivation. Consequently, MiT/TFE transcription factors are translocated into the nucleus, promoting transcription of lysosomal genes and lysosome activation. Electron microscopy analysis revealed that the number of lysosomes increased relative to that of MVBs and the level of EVs decreased after treatment with asteltoxin or rapamycin, an mTORC1 inhibitor. These findings suggest that asteltoxin represents a new type of EV inhibitor that controls MVB fate.

## Introduction

Various extracellular vesicles (EVs) are secreted by different types of cells^1^. Although EVs have been considered as a system for waste management, recent studies demonstrate that they also play important roles in intercellular communication through their complex cargo of proteins, lipids, and nucleic acids^2,3^. Secreted EVs are taken up into proximally or distally located cells and modify the properties of these recipient cells. Cancer cells secrete aberrantly large amounts of EVs that contain specific cargo molecules; that is, cancer-derived EVs are implicated in tumor progression through their contribution to construction of the tumor microenvironment and pre-metastatic niche^4–7^. Therefore, liquid biopsies using EVs for cancer diagnosis have been developed, and therapeutic strategies targeting cancer-derived EVs have been attracting increasing attention^2,8^.

Among the various types of EVs, the biogenesis of exosomes is being actively studied. Exosomes are small EVs (~ 40–150 nm diameter) and originate from intraluminal vesicles (ILVs) within multivesicular bodies (MVBs), which are formed during endosomal maturation^9^. Matured MVBs are destined to two fates: fusion with lysosomes for degradation or fusion with the plasma membrane, which leads to the release of ILVs (as exosomes) into the extracellular space^2,10,11^. The balance between targeting MVBs to lysosomes or the plasma membrane is important, and this regulation is disrupted in cancer cells. The signaling pathways regulating MVB fate and exosome formation are potential molecular targets in cancer. However, the mechanisms underlying these processes remain unclear; therefore, discovery of potent inhibitors of EVs including exosome has been limited^2,12,13^.

To date, several EV inhibitors have been reported^12,13^. Most of them inhibit Rab27A^12^, but GW4869 controls ceramide content^14,15^, and Manumycin A interferes with neutral sphingomyelinase (nSMase)^16^. In addition, EV inhibitors with different mechanisms were found through screening, including H^+^, K^+^-ATPase inhibitors such as Lansoprazole^17^, Simvastatin (which decreases lipid levels)^18^, and Sulphisoxazole (a sulphonamide antibacterial drug)^19^. However, because the potency and selectivity of these EV inhibitors was limited, novel EV inhibitors with different modes of action are eagerly desired^12,13^.

Mechanistic target of rapamycin (mTOR) is a serine–threonine kinase and a key component of mTOR complexes 1 and 2 (mTORC1/2), which play crucial roles in the control of cell growth, proliferation, and survival by monitoring diverse stimuli including growth factors, nutrients, and energy and stress signals^20,21^. Although the mTORC1 signaling pathway plays a key role in several normal physiological processes, its aberrant activation has been implicated in tumor growth, angiogenesis, and metastasis^22^. Indeed, the selective mTORC1 inhibitor rapamycin and its analogs have been developed as anticancer drugs^23^. Once activated by diverse stimuli, mTORC1 activates major downstream targets of the ribosomal protein S6 kinase 1 (S6K1) and the eukaryotic translation initiation factor 4E-binding protein 1 (4E-BP1), which play crucial roles in the regulation of mRNA translation involved in the metabolic and biosynthetic pathways^20^. Because lysosome/autophagosome biosynthesis requires coordinated transcription of many genes encoding lysosomal proteins, mTOR is a key signaling regulator of lysosome activation and autophagy^24^. In response to energy starvation, TSC2 inhibits mTORC1 activity through regulation of AMP-activated protein kinase (AMPK), a central stress-induced kinase. Lysosomes are activated in response to both nutrients and energy stressors as a result of crosstalk between the mTORC1 and AMPK signaling pathways and their coordinated control of metabolic homeostasis^20^.

In this study, fungi extracts with high structural diversity were screened using cancer cells engineered to secrete luciferase-labeled EVs. Previously, we developed a cell-based high-throughput EV quantification system by genetically labeling EV markers such as CD63 with the high-intensity luciferase NanoLuc^25^. Using this system, asteltoxin, a mitochondrial inhibitor produced by *Aspergillus ochraceopetaliformis*, was identified as an EV production inhibitor. Asteltoxin inhibited EV production at low concentrations without inducing mitochondrial damage. An asteltoxin-induced decrease in ATP level activated AMPK/mTORC1-mediated lysosome function, causing downregulation of EV production. These results indicate that asteltoxin has a unique ability to control MVB fate and suppress EV production.

## Results and discussion

### Discovery of asteltoxin, a new EV production inhibitor, from fungi extracts using cancer cells engineered to secrete luciferase-labeled EVs

**Figure 1:**
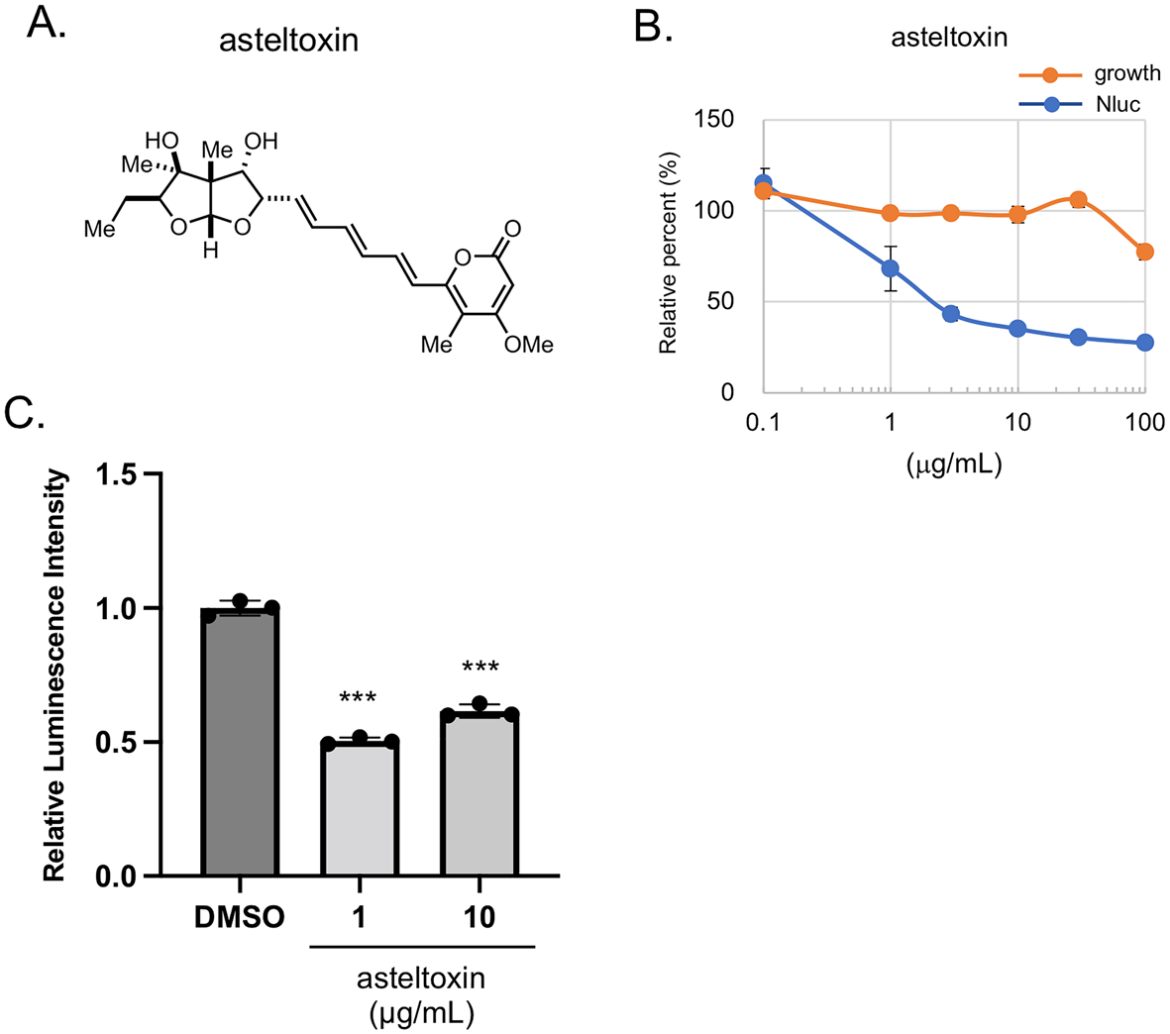
Discovery of asteltoxin, a new EV secretion inhibitor, from fungi extracts using cancer cells engineered to secrete luciferase-labeled EVs. (**A**) Chemical structure of asteltoxin. (**B**) HT29/CD63-Nluc cells were treated with different concentrations of asteltoxin for 24 h, and luminescence in the culture medium was analyzed (blue). Simultaneously, cell growth was analyzed using the WST-8 assay (orange). The relative percentages of luminescence were compared to the DMSO control (100%). (**C**) PC3 expressing CD63-Antares2 cells (PC3/CD63-Antares2) were treated with DMSO or asteltoxin at the indicated concentrations for 24 h, and luminescence in the culture medium was analyzed. Data are presented as the means ± standard deviation for three independent measurements. Statistical analysis was performed using one-way ANOVA. **P < 0.01.

We previously developed cancer cells that secrete luciferase-labeled EVs^25^, which make it possible to measure EVs produced into the culture medium in a high-throughput manner. These cells were used to screen extracts of fungi isolated from marine organisms. To exclude cytotoxic compounds, cell proliferation was simultaneously monitored. As a result of screening from 2300 of extracts library prepared from marine sponges and marine-derived microorganisms, a culture extract from the marine-derived *Aspergillus ochraceopetaliformis* 14D23-1–2 fungi was found to inhibit EV production. The bioassay-guided separation of the active EtOAc-soluble portion led to the isolation of asteltoxin (Fig. 1A), which was previously identified as a mitochondrial inhibitor. The target of asteltoxin is recognized as F1 region of ATP synthase^26^, which was supported by the crystal structure of F1-ATPase complexed with aurovertin B^27^, which is a polyketide sharing a polyene α-pyrone-type structure of asteltoxin. Asteltoxin was identified using electrospray ionization time-of-flight mass spectrometry (ESI-TOF-MS) and nuclear magnetic resonance (NMR) analyses, and these results were compared to authentic spectral data^28^. Using CD63-Nluc expressing HT29 colon cancer cells, we determined that asteltoxin inhibited CD63-positive EV production with an IC_50_ of 2.1 μg/mL but exhibited low cytotoxicity, with an IC_50_ of more than 100 μg/mL (Fig. 1B). Interestingly, other mitochondrial inhibitors, Rotenone and CCCP, suppressed cell growth at the concentrations that inhibited EV production (Supplementary Fig. S1). The reason for the difference in action observed between asteltoxin and other mitochondrial inhibitors is not known in detail at this time. However, this result suggests that asteltoxin has a characteristic mode of action other than mitochondrial inhibition.

To further investigate the mechanism of action of asteltoxin, the effect of the compound on EV secretion was examined in the prostate cancer cell line PC3, because EVs are important for the construction of the cancer microenvironment, which leads to metastasis in prostate cancer^29^. Asteltoxin decreased the luminescence level observed in the culture medium for PC3 cells expressing CD63-Antares2 (PC3/CD63-Antares2) (Fig. 1C)^30^. CD63-positive EVs from PC3 cells were 100–150 nm in size and their particle number was decreased by asteltoxin in a dose-dependent manner (Supplementally Fig. S2A and B). It was also verified to contain EV marker proteins, such as Alix and syntenin, which were suppressed by asteltoxin (Supplementally Fig. S2C). These findings suggest that the asteltoxin inhibits EV production. CD63-positive EVs inhibited by asteltoxin have the characteristics of so-called exosomes, while it is difficult to experimentally determine the origin of secreted EVs. Therefore, in this article, following the recommendations of the International Society for Extracellular Vesicles^31^, we continue to use the generic term EV hereafter.

### Asteltoxin does not induce mitochondrial damage at low concentrations

**Figure 2:**
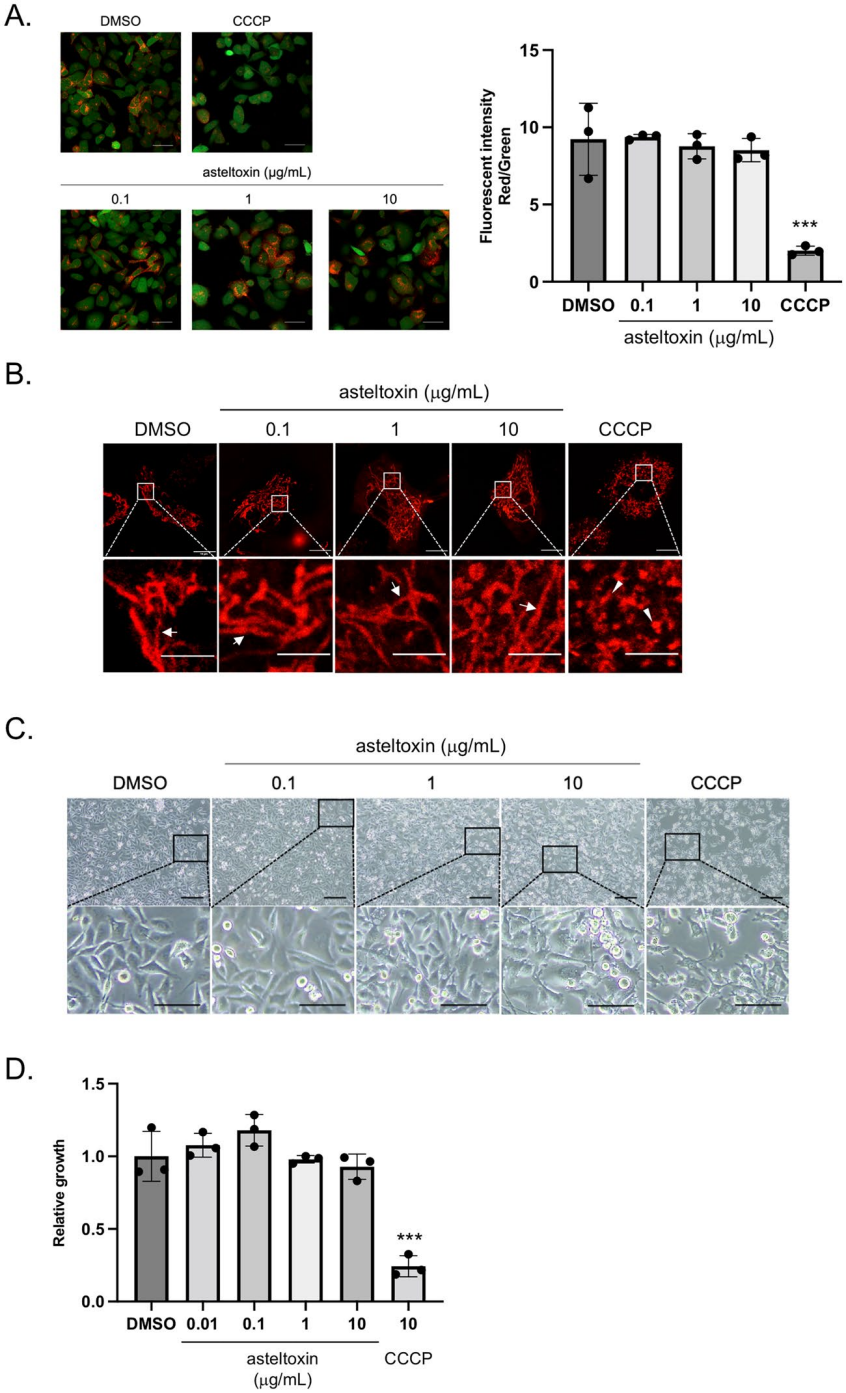
Asteltoxin does not induce mitochondria damage at low concentrations. (**A**) PC3 cells were treated with DMSO, asteltoxin (0.1, 1, or 10 μg/mL), or 10 μM CCCP for 24 h. The cells were incubated with JC-1 for 30 min and visualized using fluorescent microscopy (left panels). Fluorescence intensity with JC-1 aggregate (red)/JC-1 monomers (green) using 10,000 cells (right panel). Scale bar = 50 μm. (**B**) Cells indicated in (**A**) were stained with Mitotracker red. Images depicting fusion (arrows) or fission (arrowheads) in PC3 cells. Scale bar = 10 μm (upper panels) and scale bar = 5 μm (lower panels). (**C**) Effect of asteltoxin on the morphology of PC3 cells indicated in (**A**). Cells were photographed under a microscope. Scale bar = 200 μm (upper panels) and scale bar = 100 μm (lower panels). (**D**) Effect of asteltoxin on the proliferation of PC3 cells. PC3 cells were treated for 3 days with DMSO, asteltoxin (0.01, 0.1, 1, or 10 μg/mL), or 10 μM CCCP, and cell growth was analyzed. Data are presented as the means ± standard deviation from three independent measurements. Statistical analysis was performed using one-way ANOVA. **P < 0.01.

We examined the relevance between EV inhibition and mitochondria dysfunction. To evaluate the effect of asteltoxin on mitochondrial membrane proton pumps (MMP), PC3 cells were incubated with the fluorescent dye JC-1, which is sensitive to mitochondrial membrane potential. Even at an asteltoxin concentration that caused about 50% inhibition of EV secretion (10 μg/mL), we did not observe any significant difference in JC-1 fluorescence intensity relative to the DMSO control. By contrast, cells treated with CCCP, which was used as a positive control, exhibited significant collapse of MMP (Fig. 2A). In healthy cells, mitochondria exhibit fusion structures, but in cells with mitochondrial damage, they adopt fission structures^32^. Because mitochondrial structure dynamically changes to maintain their function, we examined the mitochondrial structure in PC3 cells. The structure of mitochondria after treatment with asteltoxin was almost identical to the fusion structure observed in the DMSO control (Fig. 2B). These results suggest that low concentrations of asteltoxin inhibit EV secretion without appreciable damage to mitochondria. Given the effects of asteltoxin on cellular events, we investigated its effects on cell phenotype. Although treatment of PC3 cells with CCCP resulted in an atrophied shape, asteltoxin did not induce dramatic changes in cell morphology (Fig. 2C). In addition, an in vitro proliferation assay showed that asteltoxin did not affect anchorage-dependent growth of these cells (Fig. 2D). Together, these results suggest that asteltoxin inhibits the EV secretion of cancer cells without causing mitochondrial damage.

### Asteltoxin decreases cellular ATP levels and activates lysosome function through AMPK-mediated mTORC1 inactivation

**Figure 3:**
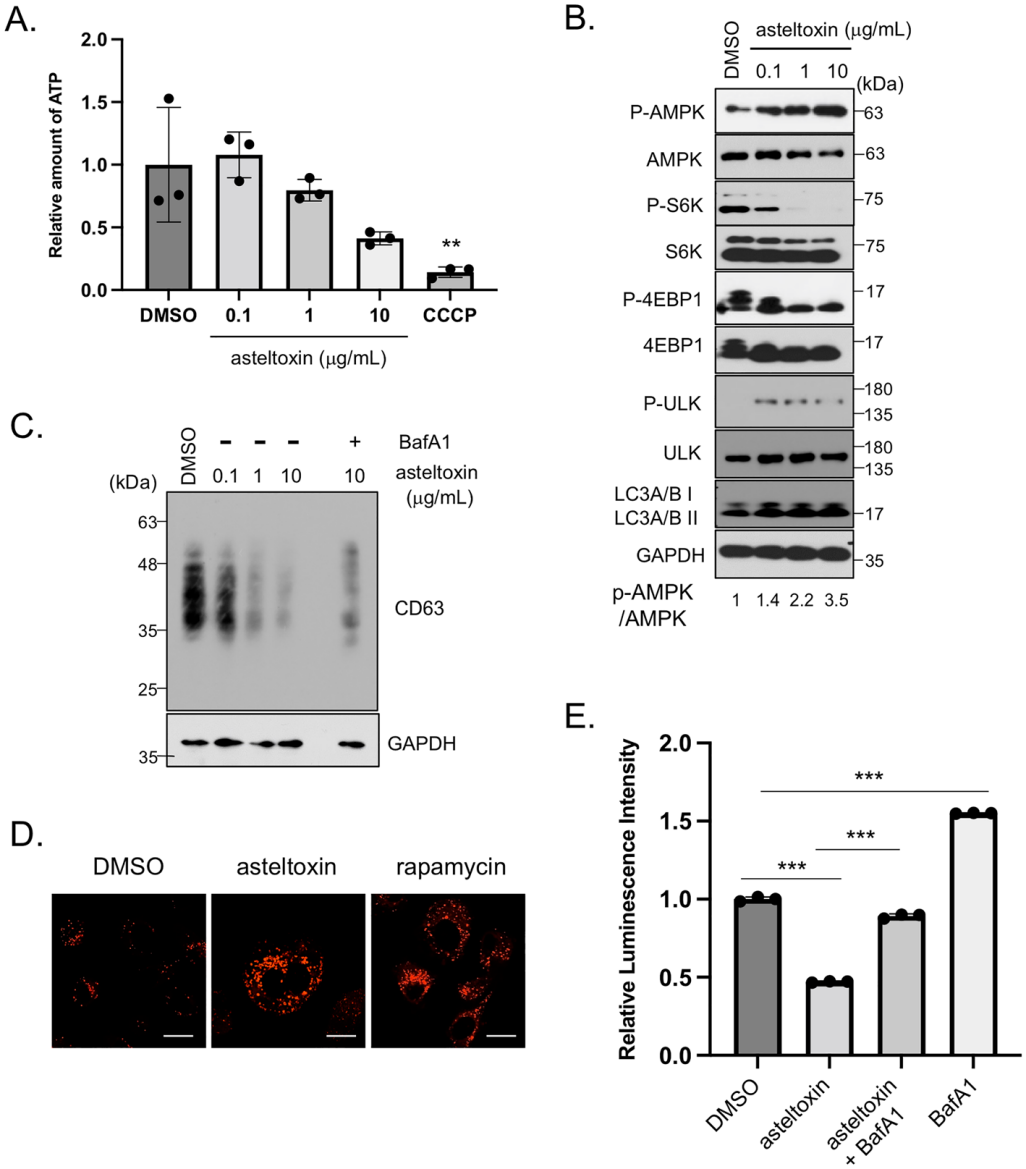
Asteltoxin suppresses cellular ATP levels and induces AMPK-mediated mTORC1 inactivation and lysosome activation. (**A**) Cellular ATP levels in PC3 cells treated with DMSO, asteltoxin (0.1, 1, or 10 μg/mL), or 10 μM CCCP for 24 h were determined. (**B**) Total cell lysates from PC3 cells treated with DMSO or asteltoxin (0.1, 1, or 10 μg/mL) for 24 h were immunoblotted with the indicated antibodies. The relative AMPK activity (p-AMPK/AMPK) levels are shown at the bottom of the panels. (**C**) Cell lysates from PC3 cells treated with asteltoxin or asteltoxin and 25 nM Bafilomycin A1 (BafA1) were immunoblotted with the indicated antibodies. (**D**) PC3 cells were treated for 24 h with 10 μg/mL asteltoxin or 200 nM rapamycin and were stained with lysotracker red DND-99. Scale bar = 10 μm. (**E**) PC3/CD63-Antares2 cells were treated with DMSO or 10 μg/mL asteltoxin with or without 25 nM bafilomycin A1 (BafA1) for 24 h, and luminescence in the culture medium was analyzed. Data are presented as the means ± standard deviation from three independent measurements. Statistical analysis was performed using one-way ANOVA. *P < 0.05 and **P < 0.01. Uncropped gel images for panels d and f are shown in Supplementary Fig. S8.

We examined the effect of asteltoxin on cellular ATP levels. As described previously^33,34^, the level of cellular ATP decreases after the addition of asteltoxin in a dose-dependent manner (Fig. 3A). These results suggest that asteltoxin treatment at the low concentrations (1 μg/mL) that are sufficient for EV inhibition results in a decrease in ATP levels to some extent, but does not induce mitochondria damage, which would lead to apoptosis. Interestingly, oligomycin, a mitochondrial inhibitor which binds to F0-ATPase^26^, also caused a similar decrease in ATP levels as asteltoxin and reduced EV production (Supplementally Fig. S3). Therefore, we hypothesized that asteltoxin influences EV secretion indirectly through ATP-related signaling. ATP depletion coupled with an increase in the AMP/ATP ratio is known to activate AMPK-mediated signaling pathways^35^. Consistently, we found that asteltoxin induces phosphorylation of AMPK at threonine 172, the major autophosphorylation site for AMPK (Fig. 3B). We next examined mTOR-related signaling, because AMPK phosphorylates the tumor suppressor tuberous sclerosis complex 2 (TSC2), thereby inhibiting mechanistic target of rapamycin (mTOR), which is involved in cell growth^36^. In PC3 cells, asteltoxin significantly suppressed the phosphorylation of S6K and 4E-BP1, critical downstream effectors of mTORC1 (Fig. 3B). AMPK activation and mTORC1 inactivation were observed at the same concentrations of asteltoxin that induced a decrease in ATP levels (more than 1 μg/mL). Taken together, asteltoxin promoted AMPK-mediated mTORC1 suppression by inducing a decrease in ATP levels. Under these conditions, we also observed the increases of ULK phosphorylation and LC3A/B (Fig. 3B), suggesting the induction of autophagy upon mTORC1 inhibition^37^. We then examined the cellular level of CD63, a marker for MVBs, because autophagosome–MVB fusion decreases EV secretion^38^. The level of CD63 was downregulated in asteltoxin-treated PC3 cells, although *CD63* transcription remained unaltered (data not shown). Interestingly, asteltoxin-induced downregulation of CD63 was appreciably restored by addition of the lysosome inhibitor bafilomycin A1 (Fig. 3C), suggesting that degradation through lysosomes determines MVB fate. Lysosome/autophagosome–MVB fusion decreases EV secretion^2^. Hence, we evaluated the impact of asteltoxin on lysosome function through the inhibition of mTORC1. Lysosome acidity, visualized by lysotracker staining, increased considerably in PC3 cells upon the addition of asteltoxin (Fig. 3D). To further verify the significance of lysosome activation in the asteltoxin-mediated suppression of EVs, we performed lysosome function rescue experiments using bafilomycin A1 (Fig. 3E). The decrease in luminescence observed in the culture medium in asteltoxin-treated PC3/CD63-Antares2 cells was significantly restored after the addition of bafilomycin A1, and the increase in luminescence observed in bafilomycin A1–treated cells was suppressed after the addition of asteltoxin. These findings suggest that asteltoxin-mediated lysosome activation is crucial for the regulation of EV secretion in cancer cells. Lysosome acidity was also observed after treatment with rapamycin, a specific mTORC1 inhibitor (Fig. 3D and Supplementary Fig. S4A). Because it was recently reported that rapamycin-mediated mTORC1 inhibition regulates EV secretion^39^, we confirmed the effects of rapamycin on EV secretion in PC3 cells. As was observed with asteltoxin, rapamycin decreased the luminescence observed in the culture medium and the number of EVs in PC3/CD63-Antares2 cells at more than 20 nM (Supplementary Fig. S4B, C). Rapamycin suppresses EVs in hepatocytes, as was observed in PC3 cells in this study, but increased numbers of EVs are observed in MEFs^39,40^. The underlying mechanism behind this difference in EV secretion caused by rapamycin is not known, but it might be due to differences in cell type. Our findings suggest that asteltoxin-mediated mTORC1 inhibition activates lysosome function and suppresses EV secretion in cancer cells.

### Asteltoxin induces nuclear localization of TFE3 and lysosome activation

**Figure 4:**
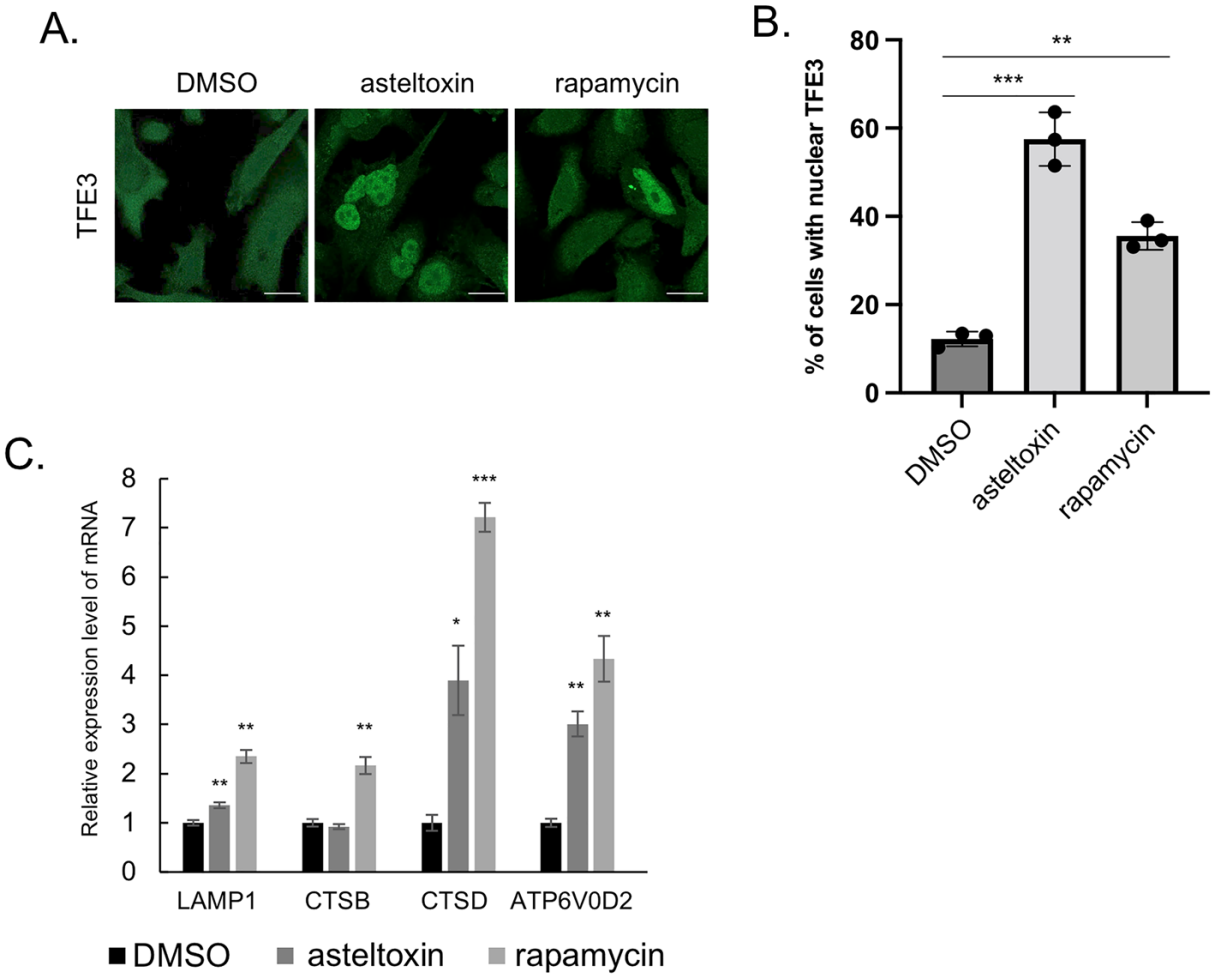
Asteltoxin induces nuclear localization of MiT/TFE transcription factor family members and promotes lysosome function. (**A**) PC3 cells were treated with DMSO, 10 µg/mL asteltoxin, or 200 nM rapamycin for 24 h and were immunostained using anti-TFE3. Scale bar = 20 μm. (**B**) Quantification of the ratio (%) of cells with nuclear localization of TFE3 (n > 77 cells, pooled from three independent experiments). (**C**) PC3 cells were treated with DMSO, 10 μg/mL asteltoxin, or 200 nM rapamycin for 24 h, and expression of lysosomal genes was analyzed. Statistical analysis was performed using one-way ANOVA. *P < 0.05 and **P < 0.01.

Because the microphthalmia family of basic helix-loop-helix-leucine-zipper transcription factors (MiT/TFE), including TFEB, TFEC, TFE3, and MITF, regulate lysosomal gene expression^41,42^, we evaluated the effects of asteltoxin on the function of MiT/TFE family members. MiT/TFE proteins are phosphorylated by mTOR or ERK2, which determines their subcellular localization; phosphorylated MiT/TFE proteins localize in the cytoplasm and bind to 14-3-3, but dephosphorylated MiT/TFE proteins localize to the nucleus and activate transcription of lysosomal genes^41,43^. Immunofluorescence analysis revealed that TFE3 was distributed in the cytoplasm, and TFEB and MITF were in both the cytoplasm and the nucleus of PC3 cells. After asteltoxin treatment, most TFE3 translocated into the nucleus (Fig. 4A and B), whereas TFEB and MITF did not exhibit considerable changes in localization (Supplementary Fig. S5). Although similar MiT/TFE protein localization changes were observed after rapamycin treatment, there were differences in the translocation ratio of TFE3 and MITF; rapamycin induced nuclear translocation of MiTF but did not affect TFE3 localization (Fig. 4A, B, and Supplementary Fig. S5). Moreover, the transcription of lysosome-related genes was upregulated in PC3 cells treated with asteltoxin (Fig. 4C). Among the lysosome-related genes tested, LAMP1, cathepsin D (CTSD), and V-type proton ATPase subunit d2 (ATP6V0D2) were significantly upregulated upon treatment with asteltoxin and rapamycin, whereas cathepsin B (CTSB) was only upregulated after rapamycin treatment. The mechanisms underlying the differences observed in the translocation of MiT/TFE proteins after asteltoxin and rapamycin treatment are currently unknown. Our results demonstrate that asteltoxin induces nuclear translocation of MiT/TFE proteins and lysosome activation via upregulation of lysosomal gene transcription, which is accompanied by suppression of mTORC1 activity.

### Effect of asteltoxin on the balance between the number of lysosomes and MVBs

**Figure 5:**
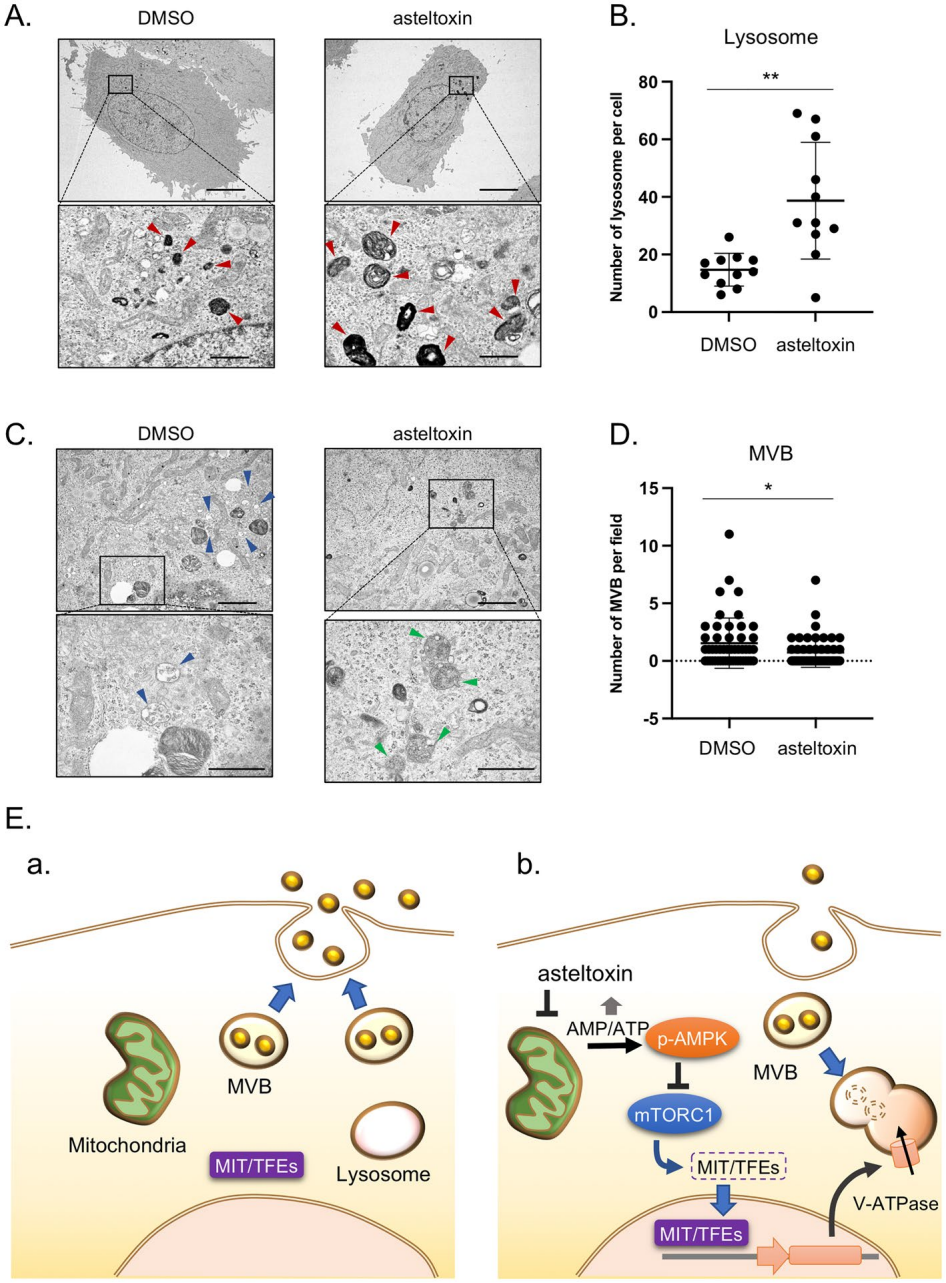
Asteltoxin controls the number of lysosomes and multivesicular bodies. (**A**) Electron microscopy images of lysosomes (red arrowheads) in PC3 cells treated with DMSO or asteltoxin (10 μg/mL) and DMSO for 24 h. Scale bar = 10 μm (upper panels) and scale bar = 1 μm (lower panels). (**B**) Quantification of the number of lysosomes per cell (n = 11 for DMSO and n = 11 for asteltoxin). MVB-lysosome fusion was counted as lysosome. Boxes represent the interquartile range, and the line inside the box represents the median value. (**C**) Electron microscopy images of MVBs (blue arrowheads) and MVB–lysosome fusion (green arrowheads) in the cells used in (**A**). Scale bar = 2 μm (upper panels) and scale bar = 1 μm (lower panels). (**D**) Quantification of the number of MVBs in the fields (n = 50 for DMSO and n = 50 for asteltoxin). The median and interquartile range are shown by bars. (**E**) Schematic model of the role that asteltoxin plays in regulating the fate of multivesicular bodies (MVBs) and controlling EV secretion. (**a**) In cancer cells, MVBs fuse with the plasma membrane and promote EV secretion. (**b**) After treatment with asteltoxin at low concentrations, the AMP/ATP ratio increases due to inhibition of ATP synthase, and this increase induces AMPK-mediated suppression of mTORC1. Inactivation of mTORC1 promotes nuclear translocation of MiT/TFE family members, thereby inducing transcription of lysosome-related genes and activation of lysosomal function. MVBs are then degraded, and the number of MVBs fused with the plasma membrane is low, resulting in suppression of EV secretion. Statistical analysis was performed using one-way ANOVA. *P < 0.05 and **P < 0.01.

Finally, we verified the role of asteltoxin in the formation of organelles in cancer cells using electron microscopy analysis. Electron microscopy analysis of PC3 cells revealed that the number of lysosomes significantly increased after asteltoxin treatment (Fig. 5A and B); by contrast, the number of MVBs markedly decreased (Fig. 5C and D). Notably, considerable levels of lysosome–MVB fusion were observed after treatment with asteltoxin (Fig. 5C, right panels). A similar result was observed after rapamycin treatment (Supplementary Fig. S6), suggesting that this phenomenon is associated with mTORC1 inactivation. Overall, these results demonstrate that asteltoxin promotes lysosome biogenesis and activity through AMPK-mTORC1 signaling, caused by ATP depletion, which results in MVB degradation and subsequent downregulation of EV secretion. A hypothetical model for asteltoxin function is presented in Fig. 5E. In cancer cells, MVBs tend to fuse with the plasma membrane to release inclusions as EVs (Fig. 5E, a). After treatment with low concentrations of asteltoxin, the AMP/ATP ratio increases through inhibition of ATP synthase, and AMPK-mediated stress signaling is upregulated without inducing mitochondria damage. Activated AMPK suppresses mTORC1 activation, thereby allowing MiT/TFE family members to translocate into the nucleus and activate transcription of lysosome-related genes including V-ATPases. Upregulation of lysosomal genes induces lysosome formation and promotes MVB degradation. Therefore, EV secretion is suppressed due to depletion of the level of MVBs (Fig. 5E, b).

Based on the mechanisms underlying EV inhibition by asteltoxin, stress-induced activation of AMPK was found to be the trigger for suppression of EV secretion. However, it is difficult to control the activation of AMPK by decreasing cellular ATP levels to a modest enough extent whereby mitochondrial damage does not occur. Hence, we attempted to inhibit EV secretion by activating AMPK in other ways. AMPK is activated by several stress signals, including amino acid and glucose starvation^36^. PC3 cells were cultured with low glucose medium. Under these conditions, AMPK was activated and the level of luminescent EVs observed was greatly suppressed (Supplementary Fig. S7A and B), even though the amount of glucose is a quarter of the normal amount given (1000 mg/L). Taking advantage of the fact that cancer cells produce ATP from glucose rather than relying on production from mitochondria, we wondered if we could inhibit EV formation specifically in cancer cells by adding 2-deoxy glucose (2-DG), a glucose analog. Addition of 2-DG induced AMPK activation in a dose-dependent manner (Supplementary Fig. S7C). Suppression of the level of luminescent EVs was also observed (Supplementary Fig. S7D). By contrast, under the same conditions, AMPK activation and EV secretion did not change in normal human keratinocyte HaCaT cells (Supplementary Fig. S7D). Although low glucose or 2-DG treatment suppresses EV secretion^44–46^, our findings provide firm evidence that stress-induced activation of AMPK by depleting glucose effectively suppresses cancer-derived EV secretion.

In conclusion, we showed that asteltoxin is a unique EV inhibitor with a potentially novel mode of action that is not categorized for currently reported EV inhibitors. Asteltoxin does not induce mitochondrial damage at low concentrations and exerts its inhibitory effect on EVs by inducing AMPK/mTORC1-mediated lysosome activation. Taken together, these results point to a unique mode of action for asteltoxin and suggest the potential usefulness of this compound as a tool for targeting EV secretion.

## Methods

### Cells and cell culture

Human colon cancer HT29 cells were obtained from the American Type Culture Collection. Human prostate cancer PC3 cells were obtained from RIKEN BioResource Research Center. Cells were cultured in the following media: HT29 in McCoy’s 5A Medium (Gibco, Waltham, MA, USA) and PC3 in RPMI Medium (Sigma-Aldrich, St. Louis, MO, USA). Each media was supplemented with 10% fetal bovine serum (FBS; Gibco). The cells were cultured at 37 °C in a humidified 5% CO_2_ chamber.

### Chemicals

Chemicals were obtained commercially: CCCP (ab141229) from Abcam (Cambridge, UK), rapamycin (AG-CN2-0025-M001) and bafilomycin A1 (BVT-0252) from Adipogen (San Diego, CA, USA), and rotenone (R0090) from Tokyo Chemical Industry (Tokyo, Japan).

### Isolation of asteltoxin from culture extracts of *A. ochraceopetaliformis* 14D23-1-2

The marine-derived *A. ochraceopetaliformis* 14D23-1-2 was isolated from an unidentified marine sponge collected at Sabang Island, Indonesia in 2014. The strain was identified as *A. ochraceopetaliformis* by Techno Suruga Laboratory (Shizuoka, Japan) based on its morphology and 5.8S rDNA sequence. *A. ochraceopetaliformis* 14D23-1-2 was cultured in rice medium (500 g of unpolished rice and 1000 mL of artificial seawater) under static conditions at 30 °C for 2 weeks. Compounds were extracted using acetone and a mixed organic solvent of acetone/MeOH/EtOAc (4:2:1), followed by combining and evaporating the organic solvents under reduced pressure to obtain a crude extract. The extract was partitioned into a water/EtOAc mixture. Using bioassays for guidance, the active EtOAc soluble portion (4.2 g) was further partitioned into an *n*-hexane/90% aq. MeOH mixture. The active 90% MeOH-soluble portion (2.5 g) was fractionated by silica gel column chromatography (eluted with CHCl_3_:MeOH) to obtain seven fractions (Fr.M1–M7). Among these fractions, Fr.M3 (698 mg, eluted with CHCl_3_:MeOH = 25:1) exhibited inhibitory activity against EV secretion. Fr.M3 was further purified using open ODS column chromatography (eluted with MeOH:H_2_O) to obtain six fractions (Fr.M3-1–M3-6). The active fraction Fr.M3-3 (289 mg, eluted with MeOH:H_2_O = 7:3) was further purified by reversed-phase HPLC [Cosmosil 5C_18_-MS-II (10 mm id × 250 mm); eluted with MeOH:H_2_O = 7:3] to obtain purified asteltoxin (20 mg). Asteltoxin was identified using ESI-TOF-MS and NMR analyses, and the results were compared with authentic spectral data^28^. NMR spectra, referenced to tetramethylsilane (TMS), were measured on an Agilent NMR system (^1^H: 600 MHz; ^13^C: 150 MHz). ESI-TOF-MS was recorded on a Q-Tof Ultima API mass spectrometer (Waters, Milford, MA, USA). HPLC was performed using a Hitachi L-6000 pump equipped with Hitachi L-4000H UV detector.

### Screening for EV inhibitors

Cell suspensions of 2 × 10^3^ cells were plated in 96-well culture dishes for 24 h, and cells were treated with samples. Two days after treatment, NanoLuc luciferase assays were performed using culture medium as described previously^25^. Cells were stained with the WST-8 reagent (Nacalai tesque, Kyoto, Japan), and proliferation was quantified by measuring absorption at 450 nm using an Immuno Mini NJ-2300 (Biotech, Tokyo, Japan). Active extracts and fractions were identified as those that could inhibit luciferase activity without suppression of cell proliferation. In the screening of 2300 extracts prepared from marine sponges and marine-derived microorganisms, we evaluated the inhibitory activities of luciferase and cell proliferation of each sample at final concentrations ranging from 1.0 to 100 µg/mL. Then, the extracts that retained more than 70% of cell viability at the concentrations that inhibited more than 60% of luciferase activity were selected as candidate samples for purifying active substances.

### Quantification of EVs

NanoLuc luciferase assays were performed as described previously using a Nivo multiplate reader (PerkinElmer, Waltham, MA, USA)^25^. For nanoparticle tracking analysis (NTA), cell supernatants were filtered through a 0.22-μm filter (MilliporeSigma, Burlington, MA, USA) and ultracentrifuged at 110,000×*g* for 70 min at 4 °C (SW41Ti rotor; Beckman Coulter, Brea, CA, USA). The size distribution and concentration of the EVs were determined using NTA with a NanoSight LM10 (Malvern Panalytical, Malvern, UK)^47^.

### Quantitative RT-PCR

Quantitative RT-PCR was performed as described previously^48^. Relative gene expressions were calculated using *GAPDH* as a control. Primer sequences are listed in Table S1.

### Western blotting

Western blotting was performed as previously described^49^. The following antibodies were used: anti–phospho-AMPK (D2D6D), anti-AMPK (D5A2), anti–phospho-ULK (D1H4), anti-ULK (D8H5), anti–phosoho-S6K (108D2), anti-S6K (49D7), anti–phospho-4E-BP1 (236B4), anti–4E-BP1 (53H11), anti-LC3A/B (D3U4C), and anti-GAPDH (14C10) from Cell Signaling Technology (Danvers, MA, USA); anti-CD63 (MX-49.129.5) was obtained from Santa Cruz Biotechnology (Dallas, TX, USA). All antibodies were used at a 1:1000 dilution.

### Immunofluorescence staining

Immunocytochemistry was performed as described previously^49^. Fluorescence was observed using a ZEISS LSM 800 with Airyscan confocal microscope (Carl Zeiss, Oberkochen, Germany).

### Photomicroscopy

Cells were plated in six-well multi-well plates; approximately 16 h after plating, cells were either left untreated or were treated with different amounts of asteltoxin or CCCP for 24 h. At the end of the treatment period, cell morphology was visualized using light microscopy and was recorded using photomicroscopy with an OLYMPUS CKX53.

### Cell proliferation assays

Cell proliferation assays were performed as previously described^48^. Cell suspensions of 4 × 10^3^ cells were plated in 96-well culture dishes for 16 h, and cells were treated with DMSO or different amounts of asteltoxin or CCCP. Two days after treatment, cells were stained with the WST-1 reagent (Merck, NJ, USA), and proliferation was quantified by measuring absorption at 450 nm using an Immuno Mini NJ-2300 (Biotech, Tokyo, Japan).

### Acidic lysosome detection

Cells on a glass-bottom dish were cultured in medium containing Lysotracker Red DND-99 (50 nM) (Thermo Fisher Scientific) for 30 min. The medium was replaced with lysotracker-free medium, and the cells were imaged using a ZEISS LSM 800 with an Airyscan confocal microscope (Carl Zeiss).

### Mitochondria detection

Cell suspensions of 1 × 10^5^ cells were plated on a glass-bottom dish, and cells were either treated with DMSO, different amounts of asteltoxin, or 10 μM CCCP for 24 h. Cells were then cultured in medium containing MitoTracker Red FM (50 nM) (Thermo Fisher Scientific) for 30 min. The medium was replaced with MitoTracker-free medium and the cells were observed using a ZEISS LSM 800 with an Airyscan confocal microscope (Carl Zeiss).

### Detection of mitochondria damage

Mitochondria depolarization was quantified using the JC-1 staining kit (Dojindo, Kumamoto, Japan). For quantification of ratio of mitochondria depolarization, cell suspensions of 1 × 10^4^ cells were plated in 96-well black plates for 24 h, and cells were either treated with DMSO, different amounts of asteltoxin, or 10 μM of CCCP for 24 h. After one day of treatment, cells were washed with Opti-MEM and stained with 2 μM JC-1 staining reagent, and mitochondria depolarization was quantified using a Nivo multiplate reader (PerkinElmer, Waltham, MA, USA). For visualization of mitochondria depolarization, cells were incubated in medium containing 2 μM JC-1 staining reagent for 30 min. The medium was replaced with JC-1 staining regent-free medium, and cells were observed using a ZEISS LSM 800 with an Airyscan confocal microscope.

### ATP assay

Amount of intracellular ATP was quantified using the Intracellular ATP Measurement kit Ver.2 (Dojindo, Kumamoto, Japan). Cell suspensions of 5 × 10^3^ cells were plated in 96-well plates for 24 h, and cells were either treated with DMSO, different amount of asteltoxin, or 10 μM of CCCP for 24 h. After one day of treatment, cells were washed with PBS, and extracted intracellular ATP using ATP extraction buffer for 5 min. Amount of intracellular ATP was quantified as luminescence using Lumat3 LB9508 (Berthord, Tokyo, Japan), and calibrated by protein concentration using BCA assay kit (Thermo Fisher Scientific).

### Electron microscopy and lysosome/MVB quantification

Cells cultured on dishes were washed in PBS and fixed for 1 h in 2.5% glutaraldehyde in 0.1 M phosphate buffer at room temperature. The cells were then slowly and gently scraped and pelleted. Pellets were washed in phosphate buffer and incubated with 1% OsO_4_ for 90 min at 4 °C. The samples were dehydrated, embedded in Spurr’s resin, and sectioned using an ultramicrotome (Leica Microsystems, Wetzlar, Germany). Ultrathin sections (50–70 nm) were stained with 2% uranyl acetate for 10 min followed by a lead-staining solution for 5 min and were observed using a JEM-1010 transmission electron microscope (JEOL, Akishima, Japan) fitted with an Orius SC1000 (model 832; Gatan, Pleasanton, CA, USA) digital camera. MVBs were identified based upon morphology and were counted; MVBs contain only discrete ILVs, whereas lysosomes contain multilamellar profiles. At least 50 MVBs were analyzed per experiment from separate cells. The minimum number of cells scored for each condition was 11.

### Statistical analysis

Data are presented as the means ± standard deviation. Statistical significance was calculated using the one-way ANOVA with Dunnett’s post hoc analysis using XLSTAT for Microsoft Excel (Redmond, WA, USA). Test results are reported as two-tailed *P*-values, where *P* < 0.05 is considered statistically significant.

## Supplementary Information

Supplementary Information.

## Data Availability

All data generated or analyzed during this study are included in this published article.
