# Supplementary material for: Asteltoxin inhibits extracellular vesicle production through AMPK/mTOR-mediated activation of lysosome function
Source: Sci Rep. 2022 Apr 23;12:6674. doi: 10.1038/s41598-022-10692-0 (PMC9035176; doi:10.1038/s41598-022-10692-0)
Supplement: Supplementary file 1 — Supplementary Information. [file 41598_2022_10692_MOESM1_ESM.pdf]

Supplemental Information:

Asteltoxin inhibits extracellular vesicle production through AMPK/mTOR-mediated activation of lysosome function

*Fumie Mitani [1,2], *Jianyu Lin [3], Tatsuya Sakamoto[1,4], Ryo Uehara[1], Tomoya Hikita[1], Takuya Yoshida[5], Andi Setiawan [6], §Masayoshi Arai [3] and §Chitose Oneyama[1, 2, 4, 7]

[1] Division of Cancer Cell Regulation, Aichi Cancer Center Research Institute, Chikusa-ku, Nagoya, Japan

[2] Department of Oncology, Graduate School of Pharmaceutical Sciences, Nagoya City University, Mizuho-ku, Nagoya, Japan

[3] Laboratory of Natural Products for Drug Discovery, Graduate School of Pharmaceutical Sciences, Osaka University, Suita, Osaka, Japan

[4] Department of Target and Drug Discovery, Nagoya University, Graduate School of Medicine, Showa-ku, Nagoya, Japan

[5] Laboratory of Biophysical Chemistry, Graduate School of Pharmaceutical Sciences, Osaka University, Suita, Osaka, Japan

[6] Department of Chemistry, Faculty of Mathematics and Natural Sciences, Lampung University, Bandar Lampung, Indonesia

[7] JST, PRESTO, Nagoya, Japan

* These authors contributed equally.

§ Corresponding author

Chitose Oneyama; coneyama@aichi-cc.jp Tel: +81-52-764-2979, Fax: +81-52-763-5233

Masayoshi Arai; araim@phs.osaka-u.ac.jp Tel: +81-6-6879-8215, Fax: +81-6-6879-8215

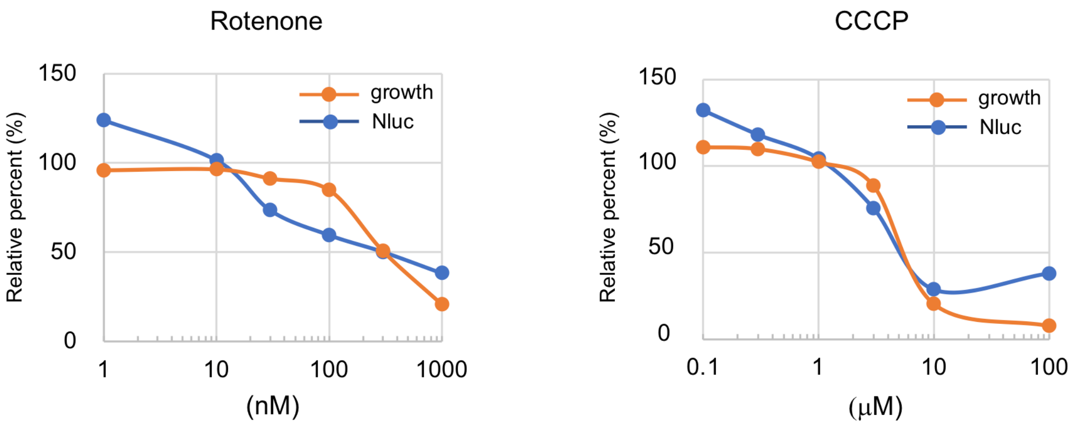

Figure S1. The effects of mitochondrial inhibitors on EV secretion and cell growth. HT29/CD63-Nluc cells were treated with different concentrations of Rotenone or CCCP for 24 h, and luminescence in the culture medium was analyzed (blue). Simultaneously, cell growth was analyzed using the WST-8 assay (orange). The relative percentages of luminescence were compared to the DMSO control (100%).

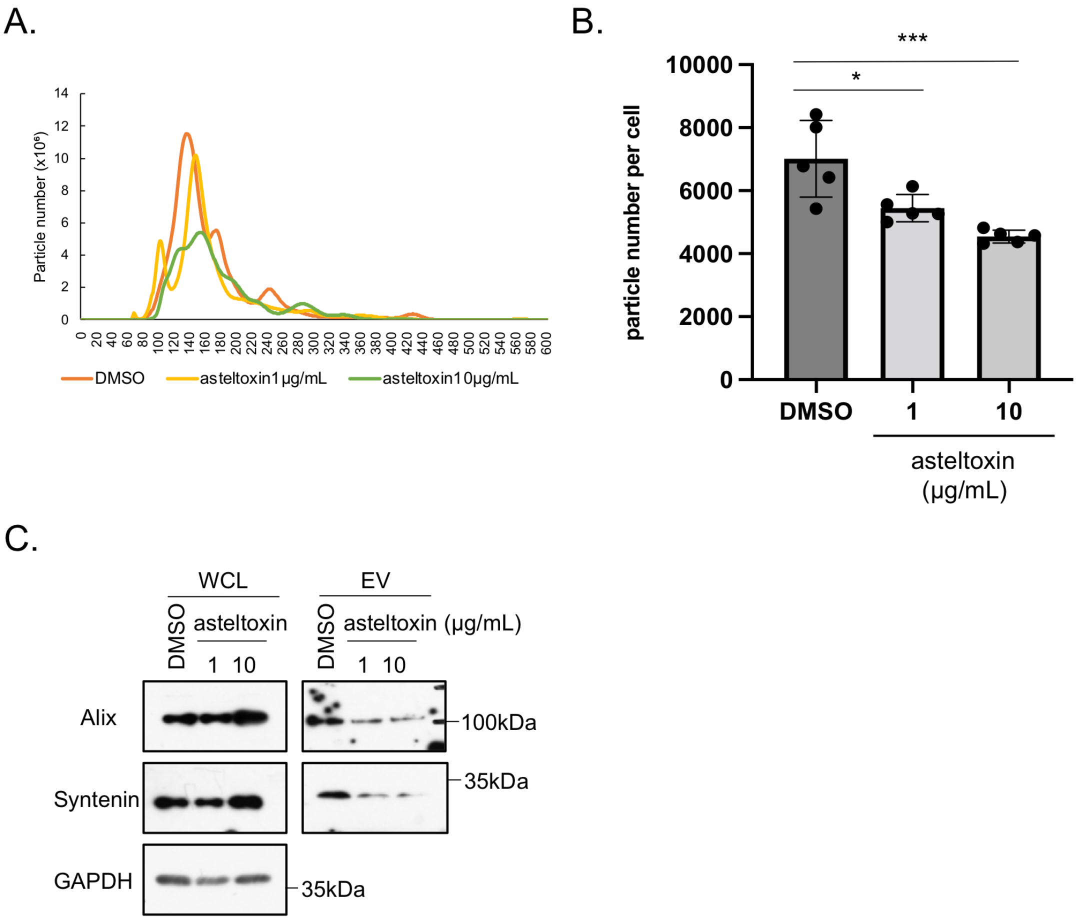

Figure S2. The effects of asteltoxin on EV secretion. (A) PC3 cells treated with asteltoxin at the indicated concentrations were examined by NTA for analysis of isolated EV particles. (B) Quantification of the EV particles in (A). (C) Total cell lysates and EVs of cells used in (A) were immunoblotted with the indicated antibodies. Data are presented as the means±standard deviation from three independent measurements. Statistical analysis was performed using one-way ANOVA. *P<0.05, ***P<0.001.

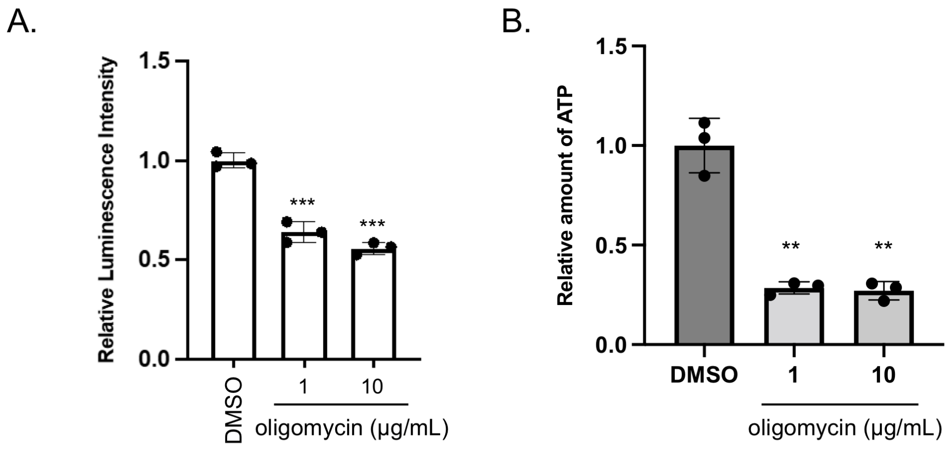

Figure S3. The effects of oligomycin on EV secretion. (A) PC3 expressing CD63-Antares2 cells (PC3/CD63-Antares2) were treated with DMSO or oligomycin at the indicated concentrations for 24 h, and luminescence in the culture medium was analyzed. (B) Cellular ATP levels in PC3 cells treated with DMSO or oligomycin (1 or 10 μg/mL) for 24 h were determined. Data are presented as the means±standard deviation for three independent measurements. Statistical analysis was performed using one-way ANOVA. **P<0.01, ***P<0.001.

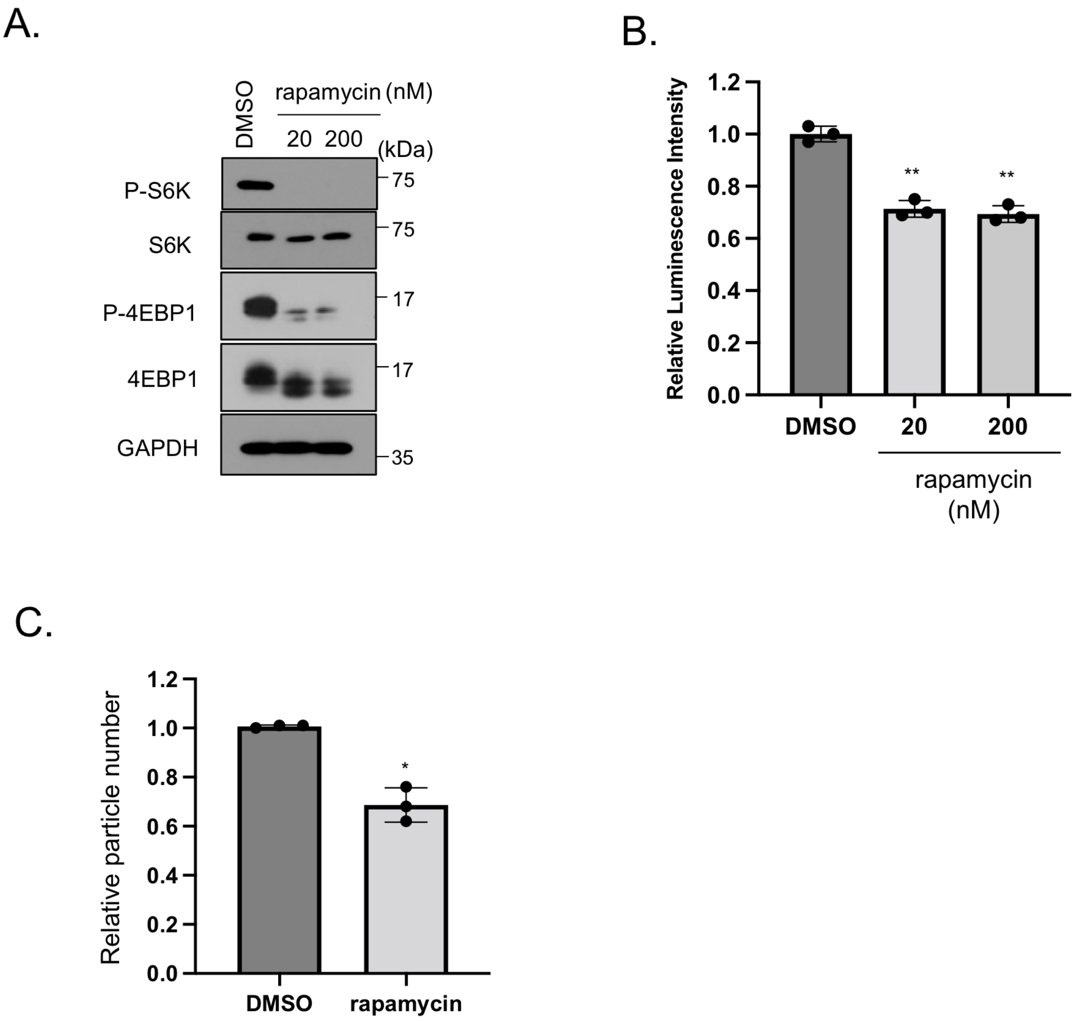

Figure S4. The effects of rapamycin on EV secretion. (A) Total cell lysates from PC3 cells treated with DMSO or rapamycin (20 or 200 nM) for 24 h were immunoblotted with the indicated antibodies. (B) PC3/CD63-Antares2 cells were treated with or without rapamycin (20 or 200 nM) for 24 h, and luminescence in the culture medium was analyzed. (C) PC3 cells used in (B) were used for EV preparation, followed by nanoparticle tracking analysis and quantification of isolated EV particles. Data are presented as the means±standard deviation from three independent measurements. Statistical analysis was performed using one-way ANOVA. **P<0.01.

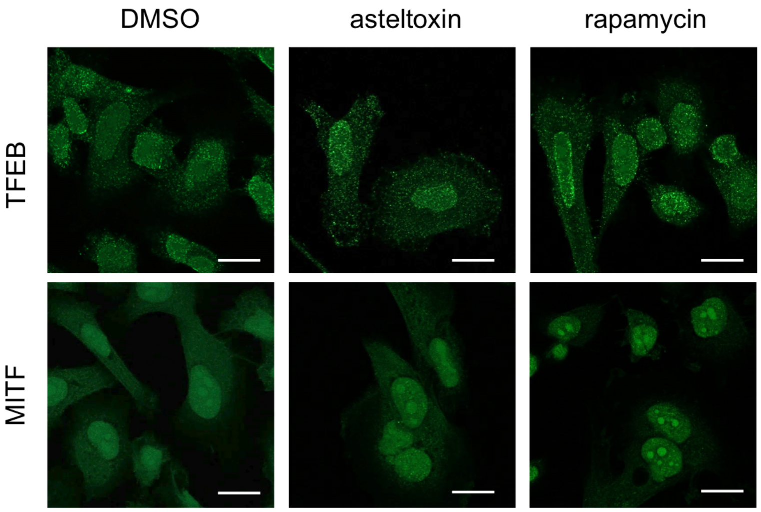

Figure S5. The effects of asteltoxin on the localization of MiT/TFE transcription factor family members. PC3 cells were treated with DMSO, 10 µg/mL asteltoxin, or 200 nM rapamycin for 24 h and were immunostained using anti-TFEB or -MITF. Scale bar=20 µm.

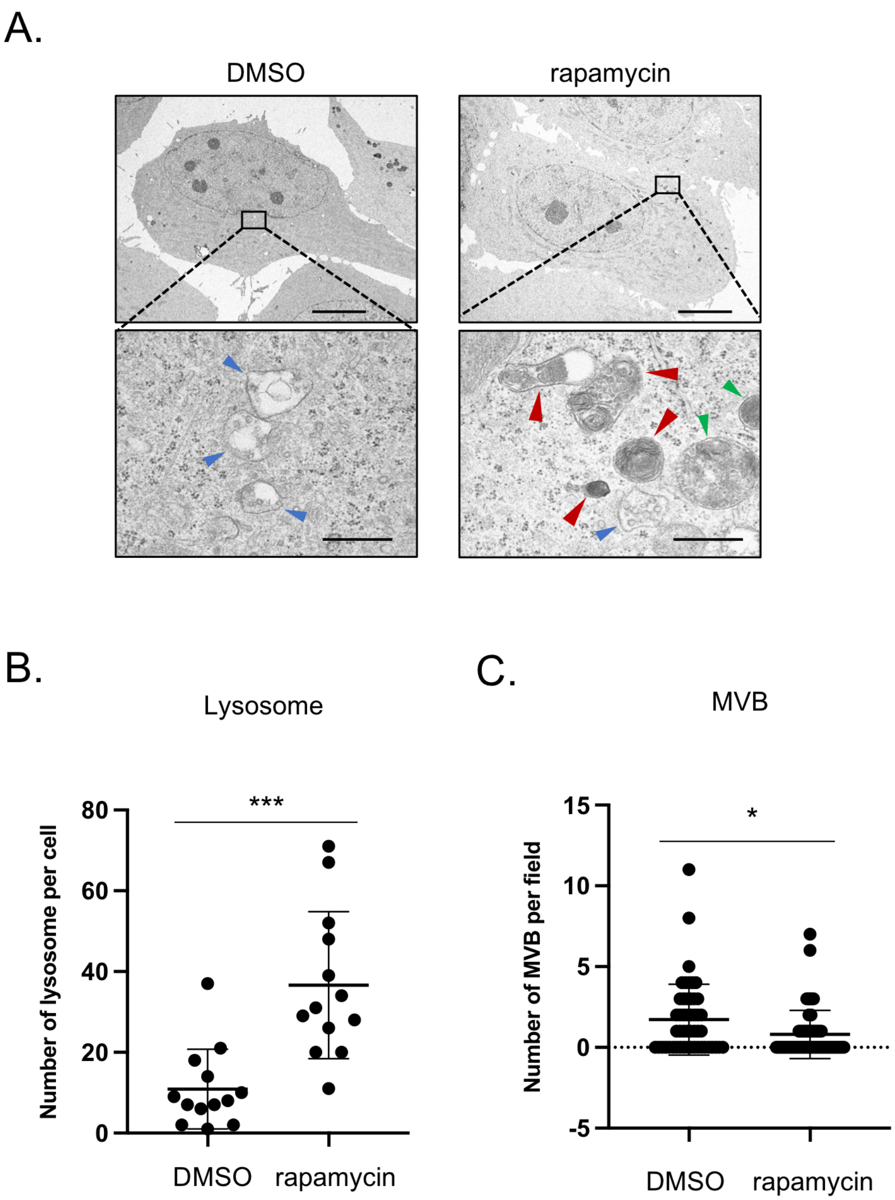

Figure S6. The effects of rapamycin on the number of lysosomes and multivesicular bodies. (A) Electron microscopy images of MVBs (blue arrowheads), lysosomes (red arrowheads), and MVB–lysosome fusion (green arrowheads) in PC3 cells treated with DMSO or 200 nM rapamycin for 24 h. Scale bar=10 μm (upper panels) and scale bar=1 μm (lower panels). Quantification of (B) the number of lysosomes per cell (n=13 for DMSO and n=13 for rapamycin) and (C) MVBs per field (n=50 for DMSO and n=50 for rapamycin). The median and interquartile range are shown by bars. Statistical analysis was performed using one-way ANOVA. *P<0.05 and ***P<0.001.

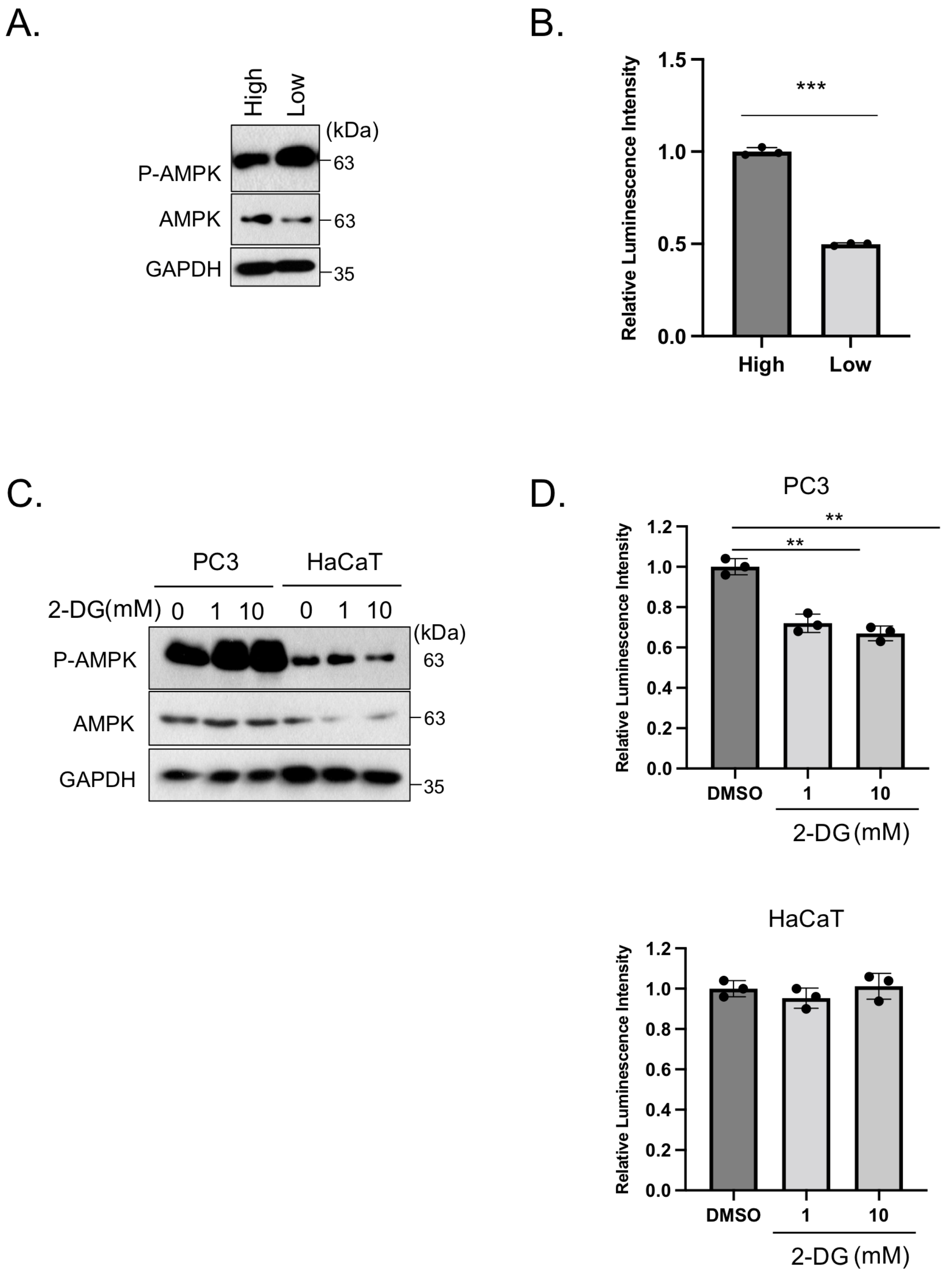

Figure S7. The effects of AMPK activation on EV secretion by measuring glucose depletion. (A) PC3 expressing CD63-Antares2 cells (PC3/CD63-Antares2) were incubated with medium containing high glucose (4000 mg/mL) or low glucose (1000 mg/mL) for 24 h. Total cell lysates were immunoblotted with the indicated antibodies. (B) Luminescence in the culture medium used in (A). (C) PC3/CD63-Antares2 cells or HaCaT-expressing CD63-Nluc (HaCaT/CD63-Nluc) cells were

treated with DMSO or 2-deoxy glucose (2-DG: 1 or 10 mM) for 24 h and were immunoblotted with the indicated antibodies. (D) PC3/CD63-Antares2 cells or HaCaT/CD63-Nluc cells were treated with DMSO or 2-DG (1 or 10 mM) for 24 h, and luminescence in the culture medium from each cell was measured and normalized to the total amount of cellular protein. Data are presented as ratios relative to the DMSO control. The results are presented as the means±standard deviation from three wells. Statistical analysis was performed using one-way ANOVA. **$P<0.01$ and ***$P<0.001$.

Figure S8. Full scan gel images.

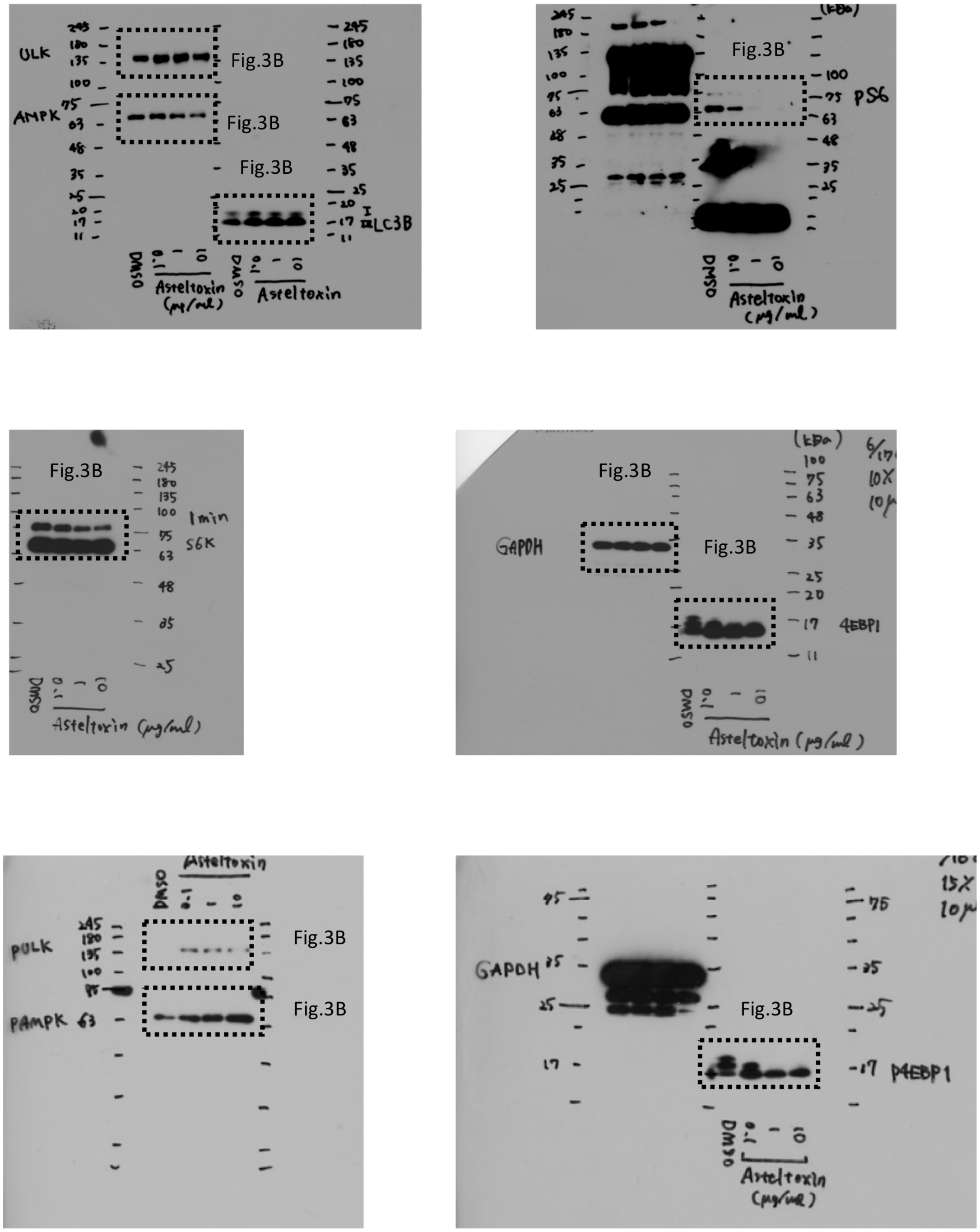

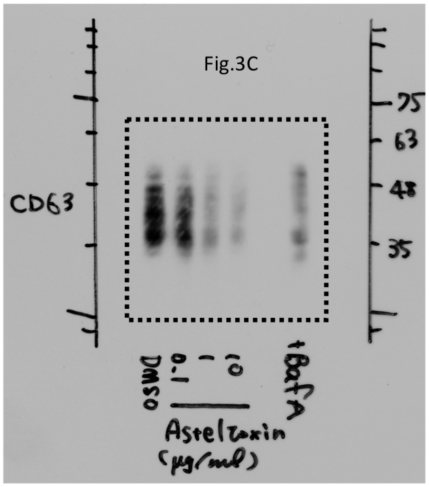

Fig.3C
CD63
75
63
48
35
DMSO
0.1
1
10
+BafA
Asteltoxin
(μg/ml)

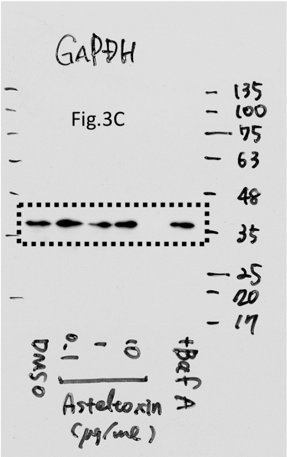

GAPDH
Fig.3C
135
100
75
63
48
35
25
20
17
DMSO
0.1
1
10
+BafA
Asteltoxin
(μg/ml)

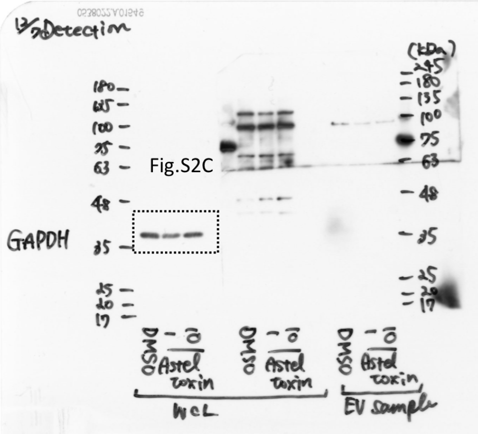

Detection
Fig.S2C
GAPDH
(kDa)
245
180
135
100
75
63
48
35
25
20
17
DMSO
1
10
Astel toxin
WCL
EV sample

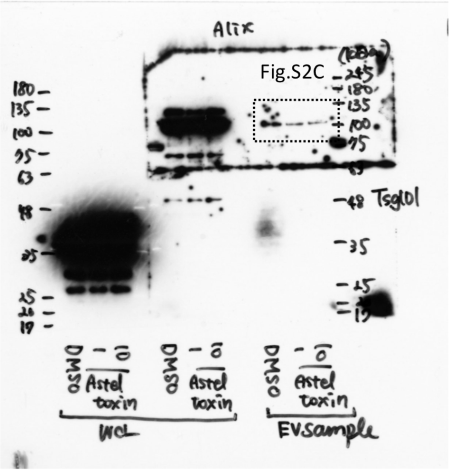

Alix
Fig.S2C
(kDa)
245
180
135
100
75
63
48
Tsg101
35
25
20
17
DMSO
1
10
Astel toxin
WCL
EV sample

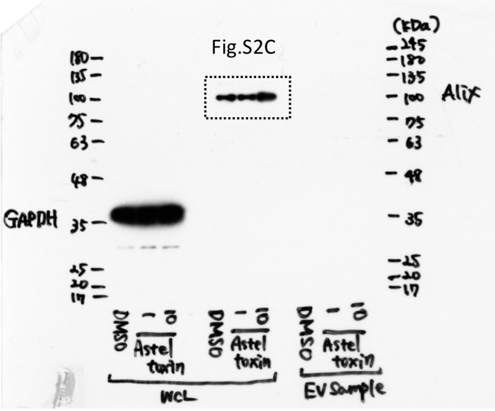

Fig.S2C
(kDa)
Alix
GAPDH
245
180
135
100
75
63
48
35
25
20
17
DMSO
1
10
Astel toxin
WCL
EV Sample

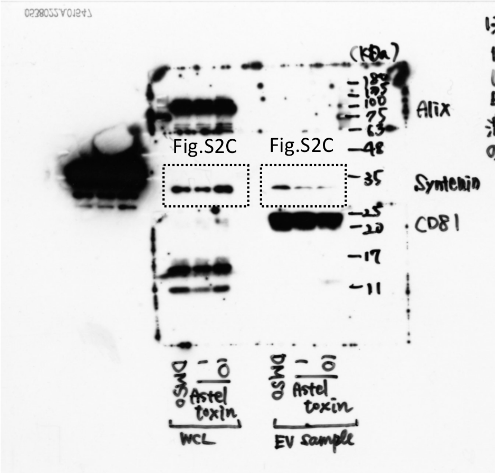

(kDa)
Alix
Fig.S2C
Fig.S2C
Syntenin
CD81
48
35
25
20
17
11
DMSO
1
10
Astel toxin
WCL
EV sample

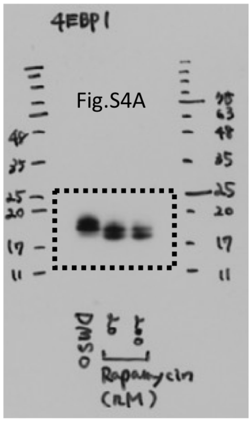
4EBP1
Fig.S4A
DMSO
Rapamycin (nM)

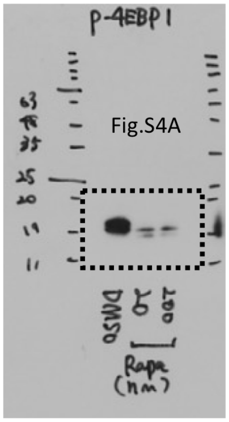
p-4EBP1
Fig.S4A
DMSO
100
Rapa (nm)

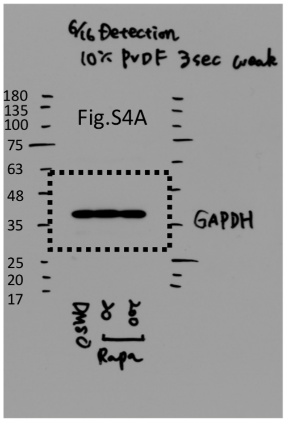
6/16 Detection
10% PVDF 3sec weak
180
135
100
75
63
48
35
25
20
17
Fig.S4A
GAPDH
DMSO
Rapa

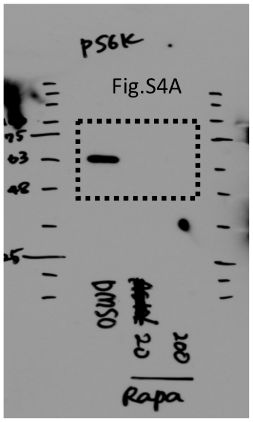
pS6K
Fig.S4A
DMSO
20
200
Rapa

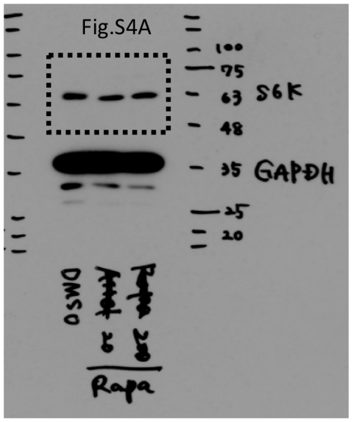
Fig.S4A
100
75
63
48
35
25
20
S6K
GAPDH
DMSO
20
200
Rapa

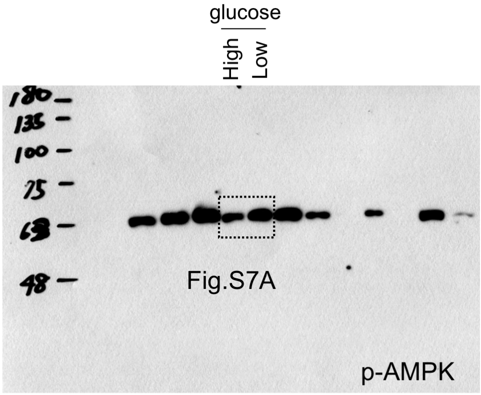
glucose
High
Low
180
135
100
75
63
48
Fig.S7A
p-AMPK

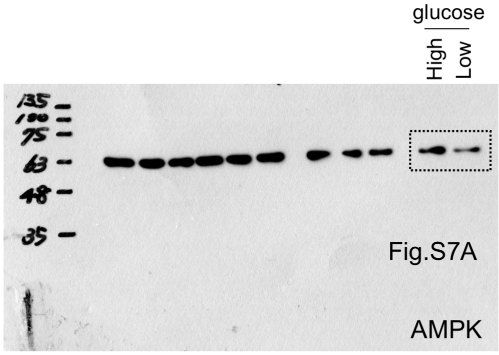
glucose
High
Low
135
100
75
63
48
35
Fig.S7A
AMPK

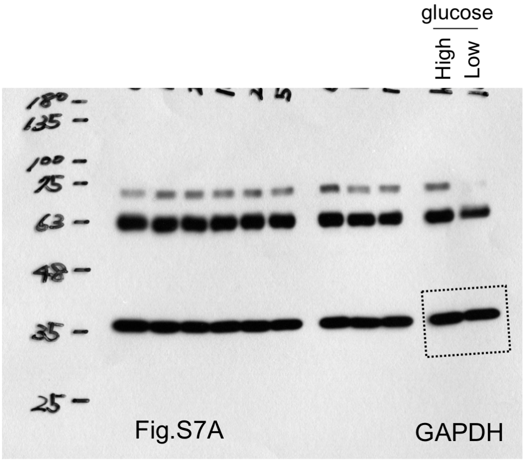
glucose
High
Low
180
135
100
75
63
48
35
25
Fig.S7A
GAPDH

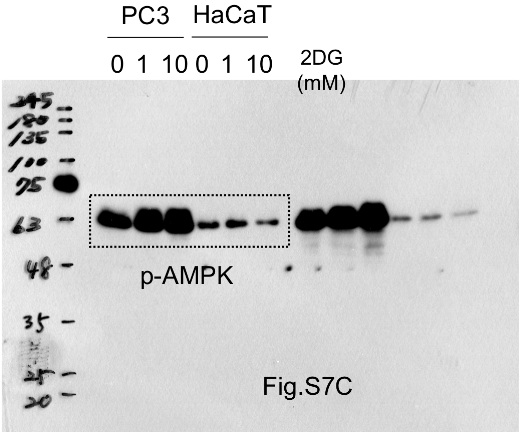
PC3
HaCaT
0 1 10 0 1 10
2DG
(mM)
245
180
135
100
75
63
48
35
25
20
p-AMPK
Fig.S7C

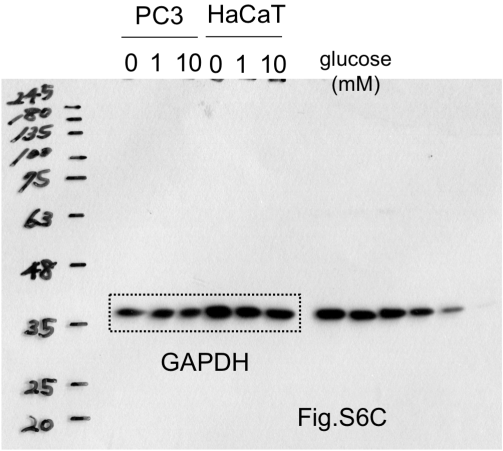
PC3
HaCaT
0 1 10 0 1 10
glucose
(mM)
245
180
135
100
75
63
48
35
25
20
GAPDH
Fig.S6C

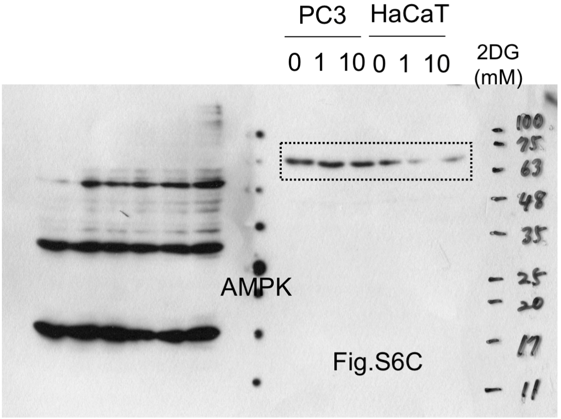
PC3
HaCaT
0 1 10 0 1 10
2DG
(mM)
100
75
63
48
35
25
20
17
11
AMPK
Fig.S6C

Table S1. Primers used in this study.

Target gene		Sequence
LAMP1	F	TTTGGCTCTGTGGAGGAGTGT
	R	GTAGGCGATGAGGACGATGAG
CTSB	F	CGTACTCCATCCCTCCCTGT
	R	TTGTATCCGTAGTGCTTGTCCTGT
CTSD	F	CCACACACACCCACACACTC
	R	GGGGAAAACCACAGAACAAAAC
ATP6V0D1	F	CGCCAAGATCGACAACTACATC
	R	CTTCACCACAGCACACAGACAC
ATP6V0D2	F	CAACCTTCGGCAAACTCTATCC
	R	CGTAATGATCCGCTACGTTCTTC
ATP6V1A	F	CTGCTGCTATCCCTGGAGCCTTT
	R	ATCTCATTTCCTCTTTCACCACATC
ATP6V1H	F	CAAAGTCAACTGGCAATCCTATCTT
	R	GCTTCTCTTCAGGGCTTCGTT
GAPDH	F	GCAAGAGCACAAGAGGAAGAGAG
	R	GAGGGGAGATTCAGTGTGGTG

LAMP1: lysosomal-associated membrane protein 1, CTSB: cathepsin B, CTSD: cathepsin D, ATP6V0D1: ATPase H+ transporting V0 subunit D1, ATP6V0D2: ATPase H+ transporting V0 subunit D2, ATP6V1A: ATPase H+ transporting V1 subunit A, ATP6V1H: ATPase H+ transporting V1 subunit H, GAPDH: glyceraldehyde-3-phosphate dehydrogenase
